# Gender Differences in the Context of Obstructive Sleep Apnea and Metabolic Diseases

**DOI:** 10.3389/fphys.2021.792633

**Published:** 2021-12-14

**Authors:** Fátima O. Martins, Sílvia V. Conde

**Affiliations:** Chronic Diseases Research Center (CEDOC), NOVA Medical School, Faculdade de Ciências Médicas, Universidade NOVA de Lisboa, Lisboa, Portugal

**Keywords:** gender, obstructive sleep apnea, metabolic dysfunction, sex hormones, chronic intermittent hypoxia

## Abstract

The relationship between obstructive sleep apnea (OSA) and endocrine and metabolic disease is unequivocal. OSA, which is characterized by intermittent hypoxia and sleep fragmentation, leads to and exacerbates obesity, metabolic syndrome, and type 2 diabetes (T2D) as well as endocrine disturbances, such as hypothyroidism and Cushing syndrome, among others. However, this relationship is bidirectional with endocrine and metabolic diseases being considered major risk factors for the development of OSA. For example, polycystic ovary syndrome (PCOS), one of the most common endocrine disorders in women of reproductive age, is significantly associated with OSA in adult patients. Several factors have been postulated to contribute to or be critical in the genesis of dysmetabolic states in OSA including the increase in sympathetic activation, the deregulation of the hypothalamus-pituitary axis, the generation of reactive oxygen species (ROS), insulin resistance, alteration in adipokines levels, and inflammation of the adipose tissue. However, probably the alterations in the hypothalamus-pituitary axis and the altered secretion of hormones from the peripheral endocrine glands could play a major role in the gender differences in the link between OSA-dysmetabolism. In fact, normal sleep is also different between men and women due to the physiologic differences between genders, with sex hormones such as progesterone, androgens, and estrogens, being also connected with breathing pathologies. Moreover, it is very well known that OSA is more prevalent among men than women, however the prevalence in women increases after menopause. At the same time, the step-rise in obesity and its comorbidities goes along with mounting evidence of clinically important sex and gender differences. Metabolic and cardiovascular diseases, seen as a men's illness for decades, presently are more common in women than in men and obesity has a higher association with insulin-resistance-related risk factors in women than in men. In this way, in the present manuscript, we will review the major findings on the overall mechanisms that connect OSA and dysmetabolism giving special attention to the specific regulation of this relationship in each gender. We will also detail the gender-specific effects of hormone replacement therapies on metabolic control and sleep apnea.

## Introduction

Obstructive sleep apnea (OSA), the most common sleep disorder, is characterized by disturbances in sleep patterns namely arousals, snoring, and recurrent episodes of cessation of breathing airflow (apnea) or airflow reduction (hypopnea) leading to excessive daytime somnolence, difficulty in concentrating, or headaches. OSA severity is classified based on the apnea-hypopnea index (AHI) and oxygen desaturation levels, which are measured in a polysomnography study. Based on AHI, the number of apneas or hypopneas per hour, OSA is clinically classified as: none/minimal: AHI < 5 per hour; mild: AHI ≥ 5, but < 15 per hour; moderate: AHI ≥ 15, but < 30 per hour; and severe: AHI ≥ 30 per hour. There are no generally accepted classifications for OSA severity based on oxygen desaturation, although reductions to not <90% are considered mild; reductions to the 80–89% range are usually considered moderate, and below 80% are severe (Hudgel, 2016).

OSA is a severe public health disease affecting 3–7% of men and 2–5% of women (Punjabi, 2008; Fietze et al., 2019). Gender differences in sleep disorders prevalence have been identified in many study-based population studies, both clinical and community-based studies, leading these disorders to be classified as a male disorder. In the first report on sleep disorder prevalence, published in the 1990s and based on the Wisconsin Cohort Study (WSCS), sleep-disordered breathing prevalence characterized as an AHI of 5 or higher was described to be 9% for women and 24% for men (Young et al., 1993). Many other studies following this one maintained the same trend or even increased the gender difference in sleep disorders, like the HypnoLaus Sleep Cohort study (Heinzer et al., 2015). This extensive study assessing the prevalence of sleep-disordered breathing in a population-based sample using the most recent polysomnographic recording techniques at the time, recorded a sleep-disordered breathing prevalence ranging from 15 to 40% in men and 4 to 35% in women, depending on the clinically defined categories (mild, moderate, and severe) or age (< or > from 60 years old) (Heinzer et al., 2015). However, the high prevalence in men compared with women based on the clinical data, which can be as high as a 90–1 male:female ratio, decreases when community-based studies are analyzed. For example, Redline and colleagues using community-based data showed that sleep apnea prevalence in women, even though lower than in men, is markedly higher than the prevalence ratio found in clinical samples which can be explained by the clinical under-recognition of symptoms of apneic activity in women (Redline et al., 1994). Also, the higher prevalence of OSA in men compared to women has been justified by cultural, anthropometric, and clinical differences between genders (Bonsignore et al., 2019; Fietze et al., 2019). For example, OSA tends to appear in older women in comparison to men, probably due to the protection provided by female-specific hormones like progesterone, which are lost when women achieve menopausal age. Also, differences in upper airway anatomy and function and differences in obesity incidence and fat distribution (more central in men and more peripheral in women) between genders justify the higher prevalence of OSA in men (Bonsignore et al., 2019). Despite the difference in reported prevalence, women represent 40 to 50% of patients coming to sleep clinics (Franklin et al., 2013). OSA typically manifests in women as a lower AHI, shorter apneic episodes, a lower proportion of supine OSA, and clustering of apnea during rapid eye movement (REM) sleep (Kapsimalis and Kryger, 2002) (Table 1). However, for the same AHI in a severe state females present higher severe oxygen desaturation justifying higher symptoms regarding reported sleepiness and more frequent recurrence to sleep clinics (Bonsignore et al., 2019). Additionally, leptin levels are higher in women which can give some protection even in an obese state, while other endocrinologic dysfunctions like hypothyroidism are highly prevalent in women, which may *per se* induce OSA (Bonsignore et al., 2019).

**Table 1:** Clinical features of obstructive sleep apnea (OSA) in females.

**Clinical features of OSA in females**
Shorter and less collapsible upper airway
Lower central chemoresponsiveness
Less respiratory drive instability
Progesterone stimulation of ventilation
Lower apneic thresholds in premenopause
Less intense snoring
Lower A/HI overall
Shorter apneic episodes
Higher frequency of REM-related OSA
Longest apneas associated with more severe arterial oxygen desaturation
Longer sleep but more disturbed

Cardiometabolic diseases, namely hypertension, metabolic diseases, cardiovascular and cerebrovascular diseases, are common in OSA, being risk factors for its development (Jean-Louis et al., 2008). Additionally, several studies showed that metabolic syndrome is highly prevalent in OSA patients and closely associated with chronic intermittent hypoxia (CIH) (Grunstein, 1996; Gambineri et al., 2003). However, the different prevalence of these metabolic alterations in men vs. women and/or aging in each gender justify, at least in part, the differences in OSA prevalence and the effects of chronic intermittent hypoxia, as well as the differences in the efficacy of the available OSA treatments in each gender. Moreover, it is fundamental to discuss the gender differences in both OSA and metabolic syndrome prevalence from the perspective of the hormonal differences between genders as well as the hormone replacement therapy (HRT) efficacy between men and women.

Altogether, this review will discuss the many conflicting aspects regarding similarities and differences between men and women in what concerns metabolic diseases and OSA prevalence. More importantly the main objective of this discussion will be to highlight how metabolism dysfunction affects OSA and vice-versa in order to better understand differences in the impact of therapies for both pathologies and the personalization of those therapies based on gender, age, body composition, and comorbidities.

## Osa-Dysmetabolism Link

Obesity, a major factor risk identified for the metabolic syndrome-related dysfunctions, has also been associated with the development and progression of OSA (Conde et al., 2014; Bonsignore et al., 2019), especially with visceral obesity, showing a link not only based on the anatomic/mechanical impact but also on the metabolic activity dysfunction (Çuhadaroglu et al., 2009). In fact, a modest decrease in body weight promotes a significant improvement in polysomnographic parameters and sleep patterns, and bariatric surgery, which promotes a huge decrease in body weight, when performed in obese patients, promotes large improvements in both OSA and diabetes-related parameters (Miras et al., 2018). Sleep apneic patients, when compared with obese controls, had a significantly higher amount of visceral fat, which correlates with O_2_ saturation and AHI levels. No correlations were found between subcutaneous fat and AHI (Vgontzas et al., 2005; Çuhadaroglu et al., 2009). However, the link between obesity and OSA is not unidirectional, as OSA can also *per se* promote body weight gain. This dual link is supported not only by weight gain but also by the multiple endocrine abnormalities that are involved in the development of fat accumulation, particularly of visceral fat, like increased insulin and/or leptin levels, reduced androgen levels, etc. (Gambineri et al., 2003) and therefore lead to and exacerbate obesity, metabolic syndrome, type 2 diabetes (T2D) (Bonsignore et al., 2013; Conde et al., 2014), and non-alcoholic fatty liver disease (NAFLD) (Jin et al., 2018).

Nevertheless, the cardiometabolic comorbidity burden is also high in nonobese OSA patients (Akahoshi et al., 2010; Gündüz et al., 2018), suggesting that other mechanisms apart from obesity contribute to the link OSA-dysmetabolism.

Using a variety of imaging techniques (computed tomography, magnetic resonance imaging, acoustic reflection, and cephalometric), several studies have also demonstrated the existence of small pharyngeal airway in patients with OSA, resulting from several syndromes or anatomical and skeletal structures alterations (Pillar and Shehadeh, 2008), which can be also a factor contributing to the link OSA-dysmetabolism. These anatomical alterations are always aggravated with increasing body fat and obesity (Pillar and Shehadeh, 2008).

### Endocrine (de)Regulation in the Link OSA-Dysmetabolism

**Figure 1:**
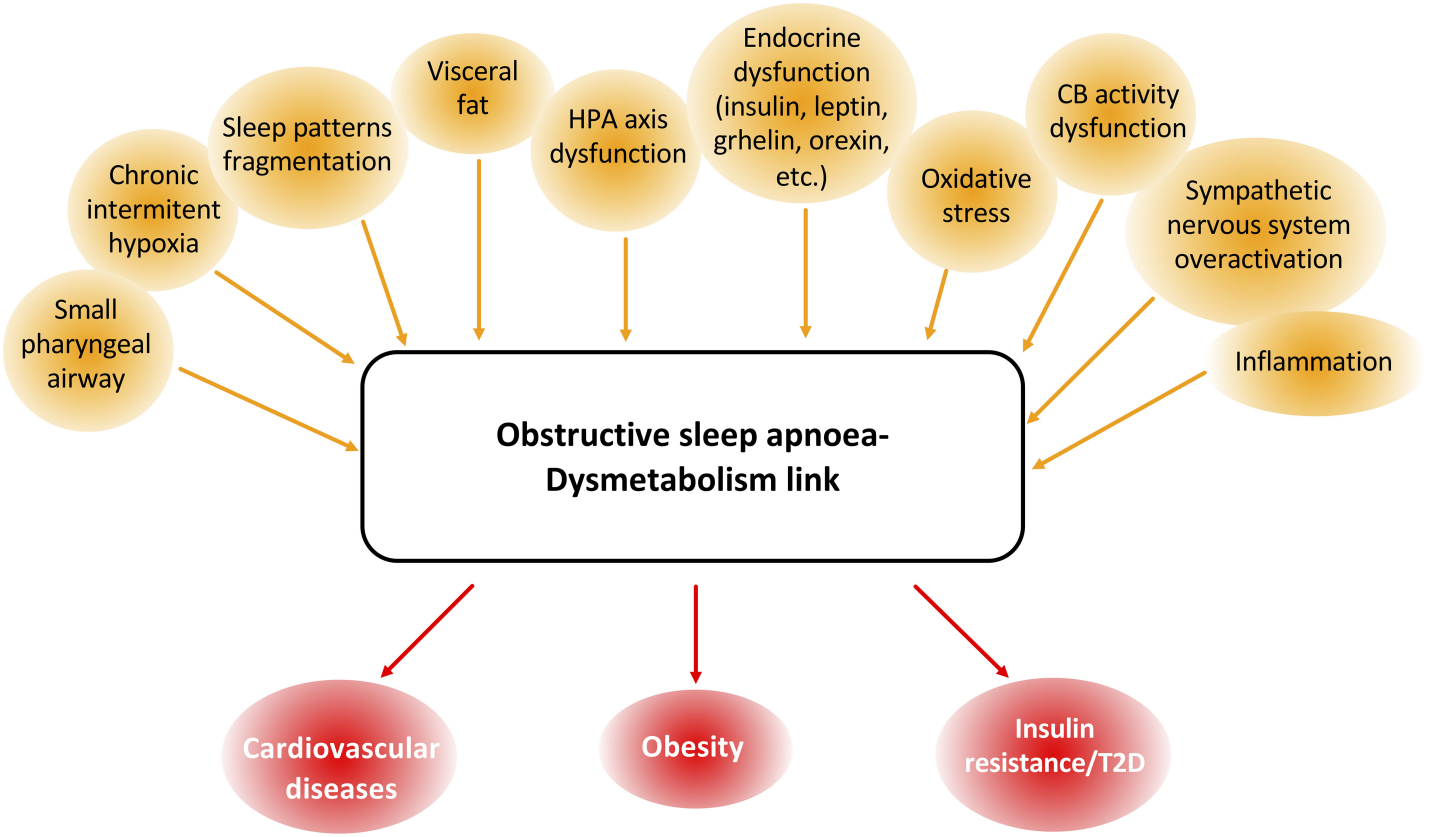
Common features and associated pathologies to obstructive sleep apnea (OSA) and dysmetabolism. OSA and dysmetabolism are associated with many common dysfunctional parameters, namely body structure and composition and endocrine, peripheral nervous system, inflammatory and oxidative stress pathways. These shared patterns between OSA and dysmetabolism are also responsible for the development and progression of many cardiometabolic dysfunctions like cardiovascular disease, obesity, and type 2 diabetes (T2D).

A growing body of epidemiological evidence supports an association between chronic sleep disorders and consequent chronic intermittent hypoxia, and the risk for obesity, insulin resistance, T2D, and many other endocrine-related diseases, showing a complex interaction between hormones and sleep disorders (González-Ortiz et al., 2000; Saaresranta and Polo, 2003; Pillar and Shehadeh, 2008) (Figure 1).

OSA may be a heterogeneous disorder in terms of its association with insulin resistance. On one hand, there are studies revealing a weak association between OSA and insulin resistance, but there are also several studies providing a strong association in favor of an independent association between OSA and insulin resistance. For example, Vgontzas et al. performed a study in which they included 14 obese men with sleep apnea and 11 BMI- and age-matched, obese non-apneic controls. The authors showed that mean fasting blood glucose levels were higher in the obese apneic compared to obese controls and that mean plasma insulin levels were also higher in those obese sleep apneic than in obese controls (Vgontzas et al., 2000). Subsequent studies showed that the association between OSA and insulin resistance was present even in nonobese subjects (Ip et al., 2002) or in mild forms of sleep apnea (Punjabi et al., 2002). This was confirmed by animal data: rats and mice submitted to CIH that mimicked OSA exhibited hyperinsulinemia and insulin resistance in the absence of obesity; pathological conditions not aggravated by the presence of increased weight (Olea et al., 2014; Shin et al., 2014; Sacramento et al., 2016; Martins et al., 2021). In addition, exhausting insulin secretion leading to beta-cell dysfunction and consequent hyperinsulinemia has been described in both animals and humans submitted to CIH or diagnosed with OSA, with special incidence in men (Polotsky et al., 2003; Pillar and Shehadeh, 2008; Wang et al., 2013). The fact that OSA is an independent risk factor for increased insulin resistance is also supported by the improvement in insulin sensitivity after 3 months of treatment with continuous positive airway pressure (CPAP) (Harsch et al., 2004; Pillar and Shehadeh, 2008). In fact, the most common treatment for moderate-severe OSA, the CPAP treatment, has been described to improve insulin sensitivity and decrease insulin levels in prediabetes (Weinstock et al., 2012; Pamidi et al., 2015) and T2D patients (Martinez-Ceron et al., 2016; Mokhlesi et al., 2016).

Another endocrine mediator that was shown to be altered in OSA is leptin. Leptin, an important adipokine secreted by the adipose tissue with known function as a satiety hormone, is a powerful respiratory stimulant (O'Donnell et al., 1999; Ribeiro et al., 2018). Leptin regulation is altered in OSA, with several studies reporting an increase in leptin levels in animal models of CIH (Polotsky et al., 2003; Olea et al., 2014) and in patients with OSA that correlated with elevated AHI (Phillips et al., 2000; Öztürk et al., 2003; Vgontzas et al., 2003; Ciftci et al., 2005; Pillar and Shehadeh, 2008), suggesting an adaptive compensatory mechanism to increase ventilation in OSA conditions. The relationship between hyperleptinemia and OSA and its metabolic comorbidities was further explored, with Polotsky et al. (2003) showing in leptin-deficient mice submitted to CIH that the increase in insulin resistance was dependent on the disruption of leptin pathways (Polotsky et al., 2003). In agreement, CPAP therapy reduced leptin levels, independently of BMI (Chin et al., 1999; Ip et al., 2000; Sanner et al., 2004; Pillar and Shehadeh, 2008). However, neither the effect of CIH on circulating leptin levels or even the levels of leptin in OSA patients nor the link between insulin resistance-OSA with the deregulation of leptin pathways was consensual. Some authors showed the absence of effect of CIH on leptin levels in animals (Briançon-Marjollet et al., 2016) and described that leptin levels of overweight subjects, children and adults, with and without OSA were not different (Yosunkaya et al., 2015; Van Eyck et al., 2017) which was consistent with the lack of effects of CPAP on leptin circulating levels (Yosunkaya et al., 2015). These differences in the role of leptin on cardiometabolic dysfunctions in OSA may indicate that leptin effects on OSA might depend on the severity and duration of the disease as well as with its interactions with obesity.

Ghrelin is another hormone that has been linked with OSA, being released by the gut, and often called the “hunger” hormone. Apart from regulating satiety and food intake, ghrelin also acts to modulate growth hormone secretion, gastric secretion and gastrointestinal motility, glucose metabolism and insulin secretion, cardiovascular functions, anti-inflammatory functions, reproductive functions, and bone formation (Gil-Campos et al., 2006). In a recent study of patients with OSA and BMI-matched control subjects, ghrelin levels were shown to be significantly higher in patients with OSA (Harsch et al., 2003; Pillar and Shehadeh, 2008). Also, Ursavas et al. (2010) described that ghrelin levels were increased in OSA patients correlating with AHI and with the Epworth sleepiness scale. Again, CPAP treatment of 2 days was sufficient to significantly reduce ghrelin levels in these patients (Harsch et al., 2003).

All this literature leads to the conclusion that disruptions in insulin, leptin, and/or ghrelin pathways contribute to metabolic dysfunction in OSA, also pointing toward possible effects of these hormones, due to their central effects, on the promotion of increased caloric intake and weight gain in patients with OSA. Also, a positive correlation between AHI and orexin levels, a central nervous system appetite regulator, may potentially explain the tendency toward weight gain in patients with OSA (Igarashi et al., 2003; Pillar and Shehadeh, 2008).

Another endocrine mechanism contributing to OSA-associated dysmetabolic states is the disruption of the hypothalamic-pituitary–adrenal (HPA) axis. It is known that repeated arousals can cause activation of the HPA axis leading to increased cortisol release (Späth-Schwalbe et al., 1998). Obesity acts as a HPA axis modulator by decreasing cortisol release, and the combination of both pathological situations can have a masking effect on the tone of this axis in OSA (Bratel et al., 1999; Vgontzas et al., 2007; Kritikou et al., 2016). However, Kritikou et al. showed that even in nonobese men or women with OSA there is an increase in cortisol levels, showing that the HPA axis-OSA association is independent of obesity. Looking at the available literature, there is controversy on the link of HPA axis and OSA since other studies fail to reveal this association (Grunstein et al., 1989; Nakamura et al., 2001; Barceló et al., 2008; Lam et al., 2009; Panaree et al., 2011) or showed a decrease in HPA activation in OSA patients (Karaca et al., 2013). The discrepancies between these studies can come from the fact that most have only assessed the involvement of the HPA axis in obese OSA patients, with a lack of data for nonobese OSA patients. In agreement with the activation of the HPA axis in OSA, CPAP had a significant effect on cortisol levels in both nonobese men and slightly obese women compared with the baseline despite CPAP not affecting the inflammation/metabolic profile of patients with OSA (Vgontzas et al., 2008; Kohler et al., 2009; Kritikou et al., 2014). Also, the administration of glucocorticoids leads to excessive cortisol levels, which then promote arousal and sleeplessness (Vgontzas and Chrousos, 2002). Likewise, patients with Cushing's syndrome, a pathological condition characterized by increased production of cortisol over a long period of time, have a high prevalence of OSA (Shipley et al., 1992). Cushing's patients complain of daytime fatigue and sleepiness resulting from the OSA condition and exacerbated by marked visceral obesity in those patients (Shipley et al., 1992; Vgontzas and Chrousos, 2002).

### Inflammation and Oxidative Stress in OSA-Dysmetabolic States

Inflammation and oxidative stress caused by CIH/OSA might be other mechanisms contributing to dysmetabolic states in OSA. There is evidence that increased levels of cytokines in circulation are involved in physiological sleep regulation and that hypercytokinemia is present in OSA (Kapás et al., 1992; Opp et al., 1992). It is well known that a positive correlation between IL-6 or TNF α plasma levels, inflammatory cytokines, and the body mass index (BMI) occurs, and this should be further evidence for the link between OSA and visceral obesity since several studies also showed that IL-6, TNF α, leptin, and insulin levels were all elevated in sleep apnea and linked with visceral fat (Vgontzas et al., 2003).

Confirming the link between inflammation and dysmetabolism-OSA, Murphy et al. (2017) found that in OSA-insulin-resistant patients the AHI correlated significantly and independently with serum levels of sCD163, suggesting M1 macrophage polarization in OSA patients. These authors also suggested that CIH/OSA promotes insulin resistance through adipose tissue inflammation (Murphy et al., 2017). These effects were supported by the results obtained in mice where it was shown that prolonged exposure to intermittent hypoxia promotes visceral white adipose tissue inflammation (Gileles-Hillel et al., 2017). However, recent data obtained by Martins et al. (Martins et al., 2021) in rats submitted to mild CIH for 35 days and to severe CIH for 15 days, did not find any alterations in the expression of pro- and anti-inflammatory cytokines within the visceral adipose tissue, demonstrating that CIH induces early metabolic dysfunction independently of adipose tissue inflammation. Therefore, we could postulate that although adipose tissue inflammation might not be the trigger for metabolic dysfunction in OSA, it could appear lately with disease progression and contribute to the maintenance and worsening of dysmetabolic states (Figure 1).

Oxidative stress induced by CIH may also play a major role in the formation of reactive oxygen species (ROS) from leukocytes, the reduction in plasma levels of nitrite, nitrate, and antioxidant capacity, increased lipid peroxidation, and increased predisposition to atherogenesis (Ramar and Caples, 2011; Bromińska et al., 2017) contributing therefore to dysmetabolic states. In fact, it is described that CIH in rats induces a whole-body oxidative status manifested by an increase in lipid peroxides and diminished superoxide dismutase (SOD) activities (Olea et al., 2014). Pointing toward a contribution of visceral adipose tissue oxidative stress to dysmetabolism in OSA, Gileles-Hillel et al. (2017) found that metabolic dysfunction after long periods of exposure to CIH is associated with increased ROS production and alterations in the electron transport chain in visceral adipose tissue (Gileles-Hillel et al., 2017). However, short exposure to mild or severe CIH during short periods did not change visceral adipose tissue oxidative stress in rats (Martins et al., 2021) suggesting that oxidative stress in the adipose tissue may develop with disease progression (Figure 1), not being involved in the triggering of metabolic dysfunction in OSA (Almendros et al., 2011; Lavie, 2015; Ferreira et al., 2020).

### Sympathetic Activation in OSA-Cardiometabolic Associated Pathologies

Sympathetic nervous system activation has been also postulated as a mechanism responsible for OSA-associated cardiometabolic comorbidities (Figure 1). OSA-associated CIH and concomitant privation of available O_2_ and increase in arterial CO_2_ results in sympathetic activation and consequent catecholamine secretion (Somers and Abboud, 1988), mechanisms that have been postulated to also be in the basis of OSA associated-cardiometabolic comorbidities, such as hypertension (Levinson and Millman, 1991; Carlson et al., 1993; Somers et al., 1995; Prabhakar and Kumar, 2010). It has been described that muscle sympathetic activity and blood and urine catecholamine levels are higher in sleep apneic patients in comparison to age- and BMI-matched controls (both norepinephrine and epinephrine) (Narkiewicz and Somers, 1999; Dempsey et al., 2010). Additionally, several studies show that chronic intermittent hypoxia can induce hypertension and sympathetic activation, both in laboratory animals (Fletcher et al., 1992; Greenberg et al., 1999; Zoccal et al., 2008; Marcus et al., 2010; Aller et al., 2020; Ferreira et al., 2020; Correia et al., 2021) and in humans (Tamisier et al., 2011). However, since sleep interruption and arousals, characteristic of the OSA condition, can also cause hypertension, some studies suggest that recurrent sleep disturbance also contributes to sympathetic overactivity and catecholamines levels increase in OSA patients, remaining to understand the specific impact of each parameter (Faraut et al., 2012; Chouchou et al., 2013; Taylor et al., 2016) on arterial pressure.

### Increased Carotid Body Chemosensitivity

**Figure 2:**
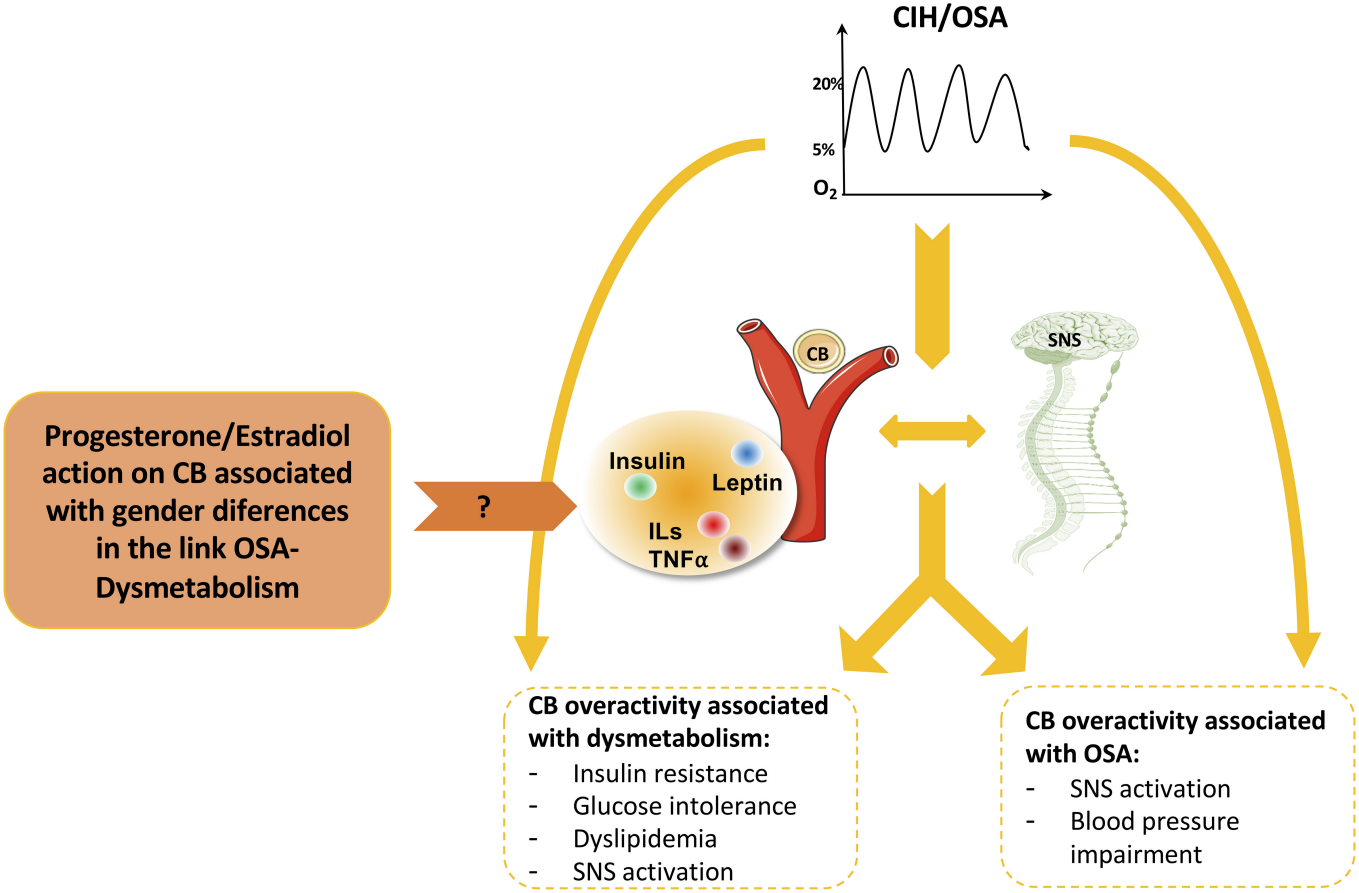
Role of carotid body (CB) and sympathetic nervous system (SNS) on the cardiometabolic pathological features associated with obstructive sleep apnea (OSA) and the possible effect of progesterone and estradiol in mediating these effects. In addition to the direct effects of OSA on dysmetabolism and cardiovascular function, CB and SNS overactivation have also an interplay in the association of CIH/OSA-dysmetabolism with impact on insulin sensitivity, glucose tolerance, lipid metabolism, and blood pressure. Many molecules have been described to have a role in this relationship by acting at the level of CB namely insulin, leptin, and inflammatory molecules, which are deregulated in conditions of cardiometabolic diseases. Based on the previous description of the existence of receptors for sexual hormones, such as estradiol and progesterone, in the CB, and knowing the impact of these hormones on the differences in the prevalence of OSA and associated dysmetabolic states between men and women we can speculate that progesterone/estradiol on the CB must have a role on the gender-specific OSA-dysmetabolism link.

The carotid bodies (CBs) are peripheral arterial chemoreceptors located at the bifurcation of the common carotid artery that classically sense and respond to changes in arterial blood O_2_, CO_2_, and pH levels. Apart from their role in the control of ventilation, it is consensual that the CBs are metabolic sensors involved in energy homeostasis (Conde et al., 2017; Conde and Guarino, 2018; Sacramento et al., 2020). CB activity is integrated in the brain *via* the CB sensory nerve, the carotid sinus nerve (CSN). Central integration of CB activity is aimed, primarily, to normalize the altered blood gases *via* hyperventilation (Gonzalez et al., 1994) and to regulate blood pressure, cardiac performance, and metabolism *via* sympathetic nervous system activation (Conde et al., 2017; Conde and Guarino, 2018). While it is quite well described that CB overactivation is responsible for the increase in sympathetic activity and blood pressure in CIH/OSA (Fletcher et al., 1992; Narkiewicz and Somers, 1999; Peng et al., 2003, 2009; Rey et al., 2004; Prabhakar et al., 2005) (Figure 1), the idea that the CBs might mediate the link between OSA and dysmetabolism emerged more recently. This idea was first supported by a study in mice submitted to CIH in where CSN denervation prevented the development of CIH-induced fasting hyperglycemia and hepatic glucose output (Shin et al., 2014). Supporting this link between the CB, dysmetabolism, and OSA, several studies demonstrated that the CB has a key role in the control of peripheral insulin sensitivity and glucose homeostasis since: (1) CSN resection prevents and reverses insulin resistance, glucose intolerance and dyslipidemia, which are pathological dysmetabolic characteristics, in hypercaloric diet rats (Ribeiro et al., 2013; Sacramento et al., 2017, 2018); (2) CSN resection prevents and normalizes the overactivation of the sympathetic nervous system associated with the intake of hypercaloric diets in rats (Ribeiro et al., 2013; Sacramento et al., 2017; Cracchiolo et al., 2019); and (3) CB activity is increased in animal models of metabolic syndrome and diabetes (Ribeiro et al., 2013; Dos Santos et al., 2018; Cracchiolo et al., 2019) and in prediabetic patients (Cunha-Guimaraes et al., 2020). Also in agreement with the role of the CB as a metabolic sensor whose dysfunction is associated with dysmetabolic states, several data showed that CB activity is modulated by hormones such as insulin (Ribeiro et al., 2013; Cracchiolo et al., 2019) and leptin (Ribeiro et al., 2018; Caballero-Eraso et al., 2019), and also by inflammation [for a review see (Conde et al., 2020)] whose levels are known to be deregulated in OSA (Ryan et al., 2005; Da Rosa et al., 2012). So, it could be postulated that CIH, by acting directly on the CB or indirectly by altering insulin secretion from the pancreas or by increasing leptin production in the adipose tissue and inflammation, leads to an increase in CB chemosensitivity promoting dysmetabolism (Figure 2).

All together we can conclude that multiple mechanisms contribute to the link between OSA and metabolic dysfunction. Additionally, we also conclude that several of these mechanisms are interrelated with each other as for example the increased CB chemosensitivity and the increased sympathetic activation in OSA. Moreover, we can also postulate that the different mechanisms may contribute differently to the link OSA-dysmetabolism depending on disease state and progression.

## Gender Differences in OSA-Dysmetabolism Link

**Figure 3:**
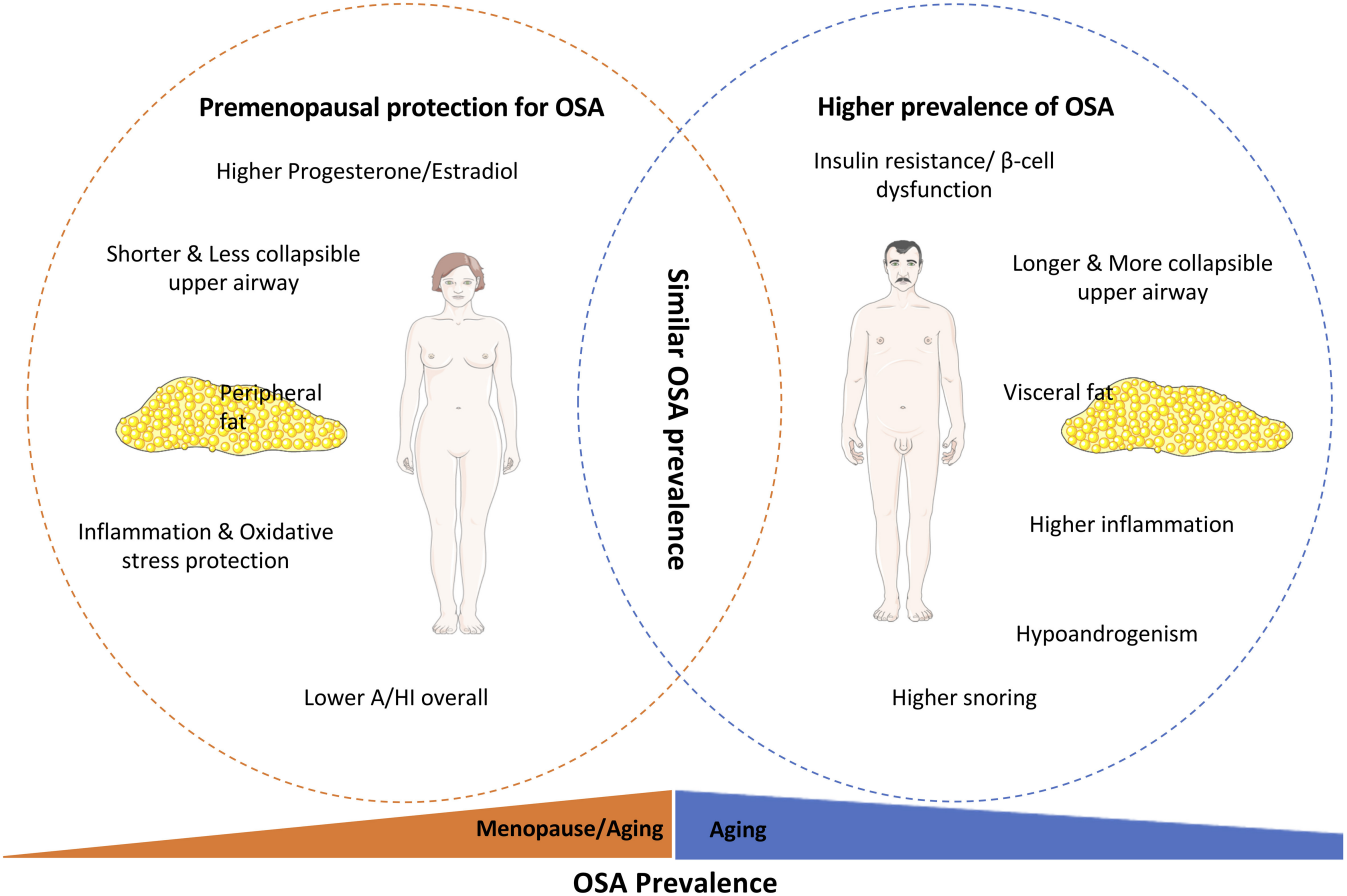
Parameters involved in the gender-specific OSA prevalence and progression. It is quite accepted that OSA is highly prevalent in men compared to women, with the latter being protected from OSA-associated dysfunctions pre-menopause. This female protection is based on the upper airway mechanical differences, inflammatory and oxidative stress protection, and higher levels of sex hormones, such as estradiol and progesterone, in women. Furthermore, the higher prevalence of OSA in men is also associated with their higher susceptibility for insulin resistance and beta-cell dysfunction and the higher visceral deposition of fat in men instead of the higher subcutaneous deposition of adipose tissue in women. With aging the prevalence of OSA increases in both females and males, leading to a similar prevalence of OSA between the genders, mainly due to the loss of sexual hormones protection in women and the similar disruption on insulin secretion and action.

In the context of the previous discussion about the mechanisms involved in the link OSA-dysmetabolism and knowing that OSA disease settlement and progression are different in men and women (Table 1), sex-specific hormones and gender differences in the OSA-dysmetabolism link are summarized in Figure 3, and will be discussed in this section.

In this scenario, we will discuss the most important sex hormones including androgens (e.g., testosterone), estrogens (e.g., 17β-estradiol), and progesterone, which are steroid hormones all derived from cholesterol (Stoffel-Wagner, 2001), by describing its general mechanisms of action as well as their specific actions in the context of OSA and dysmetabolic conditions.

Typically, these hormones can affect gene expression by binding to classical nuclear receptors, binding to steroid receptors located on cell membranes, acting on ion-gated membrane receptors, or modulating neurotransmitter receptor function (Behan and Wenninger, 2008). Estrogen exerts its effects by genomic or non-genomic mechanisms, differing in the latencies of each mechanism by minutes to hours for the former, and seconds to minutes for the latter (Brailoiu et al., 2007). In the genomic mechanism, estrogen binds to its receptor and translocates to the nucleus with a subsequent regulation of target genes (Nilsson et al., 2001). In the non-genomic mechanism, estrogen acts at the plasma membrane, activating signal transduction pathways and promoting calcium influx (Vasudevan and Pfaff, 2008). Progesterone also exerts its effect by classical genomic mechanisms and non-genomic effects of progesterone metabolites include allosteric modulation of the GABAA receptor in the brain (Smith et al., 2007). Androgen receptors are also members of the nuclear receptor superfamily and can also exert rapid, non-genomic effects *via* membrane receptors (Michels and Hoppe, 2008) (Michels and Hoppe, 2008).

Estrogen, progesterone, and testosterone are present in both women and men with different levels between gender and during their lifetime. Testosterone circulating levels are typically higher in men than in women reaching their peak at 18–40 years old in male humans (Smith et al., 1992; Bhasin et al., 2006). Serum estrogens levels are low and similar between genders at birth and higher in women in adult life (Amateau et al., 2004).

### Sex Hormones and OSA

Sleep patterns differ between men and women, with women reporting sleeping longer, but with more disturbed sleep and waking up more times than men (Figure 3) (Orff et al., 2014). Hormonal changes associated with gender-specific hormonal composition, the menstrual cycle, pregnancy, postpartum, oral contraceptive use, psychological disturbances (depression, anxiety, etc.), and/or lifestyle are on the basis of male vs. female differences and specific OSA-dysmetabolism relationship in women (Krystal et al., 1998; Krystal, 2003).

Some controversy can be found when the question of which sex hormone is the most effective in the regulation of breathing rises. In fact, for many years, hyperventilation in human pregnancy in the luteal phase of the menstrual cycle has been associated with elevated progesterone levels (Behan and Wenninger, 2008). What is known is that during the menstrual cycle, female sex hormones affect respiratory control and upper airway stability, and these hormones are described to be protective and responsible for the lower rates of OSA in women in comparison to men (Figure 3) (Netzer et al., 2003). In fact, women diagnosed with OSA have lower levels of progesterone, estradiol, and 17-OH progesterone (Netzer et al., 2003). Also, previous polysomnographic studies showed that the prevalence and severity of OSA are increased in postmenopausal vs. premenopausal women independently of body weight (Dancey et al., 2001). Progesterone is an important breath stimulant hormone therefore it has been suggested that the decrease in progesterone levels in menopause must be involved in the increase in OSA prevalence in the postmenopausal period (Figure 3) (Orff et al., 2014). Nonetheless, administration of synthetic progestin to healthy men increased minute ventilation as well as the hypoxic and hypercapnic ventilatory responses (Skatrud et al., 1978). Thus, it is clear that progesterone stimulates breathing in both men and women but the exact mechanism is unknown with some studies arguing that respiratory effects of progesterone are mediated by progesterone receptors-containing cells in the hypothalamus, by modulating the release of neuromodulators such as serotonin and/or binding and modulating GABA receptors function (Behan and Wenninger, 2008). Moreover, Boukari et al. (2016) showed that administration of siRNA against membrane progesterone receptor-β, but not -α, in dorsal brainstem increased apnea frequency, suppressed the response to hypoxia in male and female mice, and reduced by ~50% the response to hypercapnia in male and female mice (Boukari et al., 2016). Additionally, the authors showed that females exhibited a higher ventilatory response to hypoxia and hypercapnia than males, effects abolished by membrane progesterone receptor-β siRNA, but not -α, demonstrating that membrane progesterone receptor-β in the dorsal brainstem establishes sex-specific chemoreflex responses and reduces apnea frequency in adult mice (Boukari et al., 2016). Another mechanism by which sex hormones can act to modulate breathing and increase the response to hypoxia is by acting on the CB (Figure 2) (Joseph et al., 2013). It was shown that when ventilation—basal and in response to hypoxia—and CB neural output were evaluated in castrated male cats before and after 1 week of a placebo, estrogen, progesterone, or estrogen plus progesterone treatment, animals receiving progesterone alone or in combination with estradiol had higher ventilation and CB neural output responsiveness than animals receiving placebo or estrogen (Hannhart et al., 1990). However, the slope of the correlation between ventilation and CB neural output was higher in estrogen-treated than in the placebo animals, suggesting a combined action of estrogen and progesterone in central and CB sites, respectively, to modulate breathing (Hannhart et al., 1990). In agreement, progesterone also increased CB response to hypoxia in rats and mice (Joseph et al., 2006, 2013), effects consistent with the presence of progesterone receptors in the CB (Joseph et al., 2006, 2013). The CB-mediated effects of progesterone on ventilation were confirmed in newborn animals since the CB responses to hypoxia and nicotine were dramatically decraesed by mifepristone, a progesterone and glucocorticoid receptor antagonist with weak anti-androgenic activity (Joseph et al., 2012).

More recently, the effect of progesterone on arterial blood pressure, respiratory chemoreflexes, and oxidative stress in the central nervous system was studied in ovariectomized female rats submitted to room air and to 1 week of CIH (7 days, 10% O_2_, 10 cycles h-1, 8 h day-1) (Joseph et al., 2020). The authors found that while progesterone administration does not prevent hypertension elicited by CIH, it normalizes respiratory control, and reduces cerebral oxidative stress, suggesting that modulation of progesterone levels can attenuate the consequences of sleep apnea in menopausal women. Although it remains to be established if these effects of progesterone on CIH female rats could be mediated by the CB or other central structures.

Regarding the effect of estrogen on ventilation little is known but it has been implicated in the control of breathing since it has been associated with OSA and estrogen or combined estrogen-progesterone therapy have therapeutic effects over ventilation regulation (Shahar et al., 2003). In fact, as stated before and described by Hannhart et al. (1990) while estradiol did not change ventilation and CB neural output in cats, the correlation between ventilation and CB neural output was higher in estrogen-treated than in the placebo animals, suggesting an interaction between progesterone and estradiol in breathing control.

Finally, testosterone is implicated in OSA and breathing control due to the high OSA prevalence and low levels of testosterone in middle-aged men (Young et al., 2003) and also due to the fact that testosterone administration in women promoted increase in ventilation during wakefulness and increased ventilatory sensitivity to CO_2_ during sleep (Ahuja et al., 2007). These hormones have synergistic effects over ventilation since for example administration of testosterone in healthy men and women can upregulate progesterone receptors in the brain and therefore increase minute ventilation and hypoxic responses (Simpson et al., 2002). From the major structures involved in the control of breathing, we must highlight the CB and the brainstem due to the presence of sex hormones receptors on these sites and alteration of the activity of these structures when changes in hormones levels occur (Behan and Wenninger, 2008).

### Sex Hormones and OSA-Dysmetabolism Link

Dysmetabolic states prevalence differs between genders, not only on the higher prevalence of obesity in men, especially visceral obesity, but also in the prevalence of other pathological states as insulin resistance, T2D, etc. (Goodpaster et al., 2003; Gautier et al., 2013). The impact of visceral obesity on OSA incidence and severity can also explain the higher prevalence of OSA in men, the gender that is more affected by the deposition of fat in the abdominal sites (Figure 3) (Gautier et al., 2013). However, women with OSA exhibit stronger correlations than men between OSA and diabetes, hypertension, age, BMI, and waist-to-hip ratio, with men demonstrating a slightly stronger association with metabolic syndrome (Fietze et al., 2019). These stronger correlations in women than men for OSA and hypertension have been found by other authors, although with no gender differences regarding BMI, snoring, and diabetes (Huang et al., 2018).

Reduced testosterone levels represent an independent cardiovascular risk factor in the general male population and are intimately correlated with obesity, insulin resistance, and visceral fat deposition (Mårin et al., 1992; Mårin, 1998). Testosterone and other androgens stimulate lipolysis in the adipose tissue by inhibiting lipoprotein lipase activity, particularly at the visceral level (Björntorp, 1996; Gambineri and Pasquali, 2000). In fact, men with low levels of testosterone present enlarged visceral fat that can be completely reversed with testosterone hormone replacement (Gambineri and Pasquali, 2000). The sex-specific difference of the impact of OSA on glucose metabolism remains mostly unexplored.

Interestingly, increased oxidative stress, low testosterone, and male sexual dysfunction are shared outcomes between middle-aged rats and CIH exposed young male rats, these results suggesting that CIH-induced oxidative stress is one of the primary factors involved in CIH-induced reproductive aging in males.

Another factor that can explain the low levels of OSA and its link with cardiometabolic comorbidities in pre-menopause women and the increase in their prevalence post-menopause is the effect of sex hormones, particularly progesterone at the CB (see Section Sex Hormones and OSA). Progesterone and estradiol receptors are present in the CB and the activation of progesterone receptors within the CB was shown to decrease CB response to hypoxia in animals—mouse, rat, and cat (Hannhart et al., 1990; Joseph et al., 2006, 2013). In agreement with the effect of sex hormones on ventilation and on CB, the reduction of ovarian hormones, either by ovariectomy or aging, attenuates the ventilatory response to hypoxia (Fournier et al., 2015). Knowing that OSA associated cardiometabolic pathologies run with altered CB activity (Conde et al., 2014; Iturriaga, 2018) and knowing the impact of these hormones on the differences in the prevalence of OSA, we might speculate that progesterone/estradiol on the CB must have a role on the gender-specific OSA-dysmetabolism link.

Also, loss of estrogens after menopause are associated with obesity, cardiovascular diseases, and the increase in inflammatory markers, giving to menopausal women a similar prevalence of sleep apnea as in men (Vgontzas and Chrousos, 2002). The lower OSA prevalence in women seems to be also associated with an inflammatory profile less severe in women than in men, although women suffering from OSA are more obese than men (Figure 3) (Bonsignore et al., 2012). The stronger elevation of inflammatory markers in men than in womenis consistent with the greater degree of sleepiness observed in men.

Women with polycystic ovary syndrome (PCOS), a condition associated with hyperandrogenism and insulin resistance, have increased sleep-disordered breathing and daytime sleepiness than controls, supporting again the impact of insulin resistance in OSA pathology (Dunaif, 1997). Given that PCOS is associated with insulin resistance and central obesity, these data reinforce the hypothesis that obesity and/or insulin resistance is at the basis of the pathologic mechanisms leading to OSA in women (Van Cauter and Spiegel, 1997).

## Therapies on Metabolic Control and OSA

In this section a detailed discussion about the therapeutics for control of OSA can be found as well as their impact of those same applications over metabolic control with special attention to CPAP, hormone replacement therapy (HRT) and other physical and nutritional interventions.

### CPAP Treatment for OSA and Dysmetabolism

The most widely used therapy for OSA treatment is CPAP. The beneficial effects of CPAP appear to be partial as previous studies have shown that CPAP does not, in short-term use, affect the inflammation, insulin resistance, or metabolic profile of patients with sleep apnea (Saini et al., 1993; Smurra et al., 2001). However, in long-term and high adherence CPAP treatment, beneficial effects on insulin levels and glucose tolerance have been shown by some authors (Brooks et al., 1994). This is important given the independent association of insulin resistance and inflammation with cardiovascular morbidity and mortality (Kritikou et al., 2016). In a previous meta-analysis, CPAP significantly improved insulin resistance in non-diabetic patients with moderate to severe OSA, without significant changes in BMI or fasting blood glucose (Yang et al., 2013). Another study demonstrated a significant increase in insulin secretion and decrease in circulating leptin, total cholesterol, and LDL cholesterol by CPAP treatment, showing the protective effects of CPAP treatment against the development of metabolic syndrome in OSA patients (Çuhadaroglu et al., 2009). Similar to OSA prevalence, gender impacts also on CPAP effectiveness since the requirements for this therapy are higher for men than women for the same OSA severity (Yukawa et al., 2009; Jayaraman et al., 2011).

### HRT and Its Impact on OSA and Metabolic Dysfunction

The positive effects of HRT on OSA and/or metabolic dysfunction raises some concerns since there is controversy surrounding the studies found in the literature. In fact, it has been assumed that the beneficial or adverse effects of HRT will depend on the patient's comorbidities, hormone type or combination of hormones, route of administration, and dosage (Fineberg, 2000). For example, the impact of HRT on promoting an increase in the levels of triglycerides, based on the administration of conjugated estrogens, can be avoided using transdermal estrogen or oral estradiol (Fineberg, 2000). Also, few long-term risks for the development of diabetes mellitus have been shown for women receiving postmenopausal HRT (Mauvais-Jarvis et al., 2017). In fact, large controlled trials suggested that HRT using estrogens in menopausal women reduces the incidence of type 2 diabetes (Espeland et al., 1998; Kanaya et al., 2003; Margolis et al., 2004; Salpeter et al., 2006; Manson et al., 2013). These and many other studies have demonstrated that HRT can have beneficial effects on body weight, insulin sensitivity, carbohydrate or lipid metabolism (Mauvais-Jarvis et al., 2017). In one study combined HRT treatment prevented, for example, an increase in abdominal fat in menopausal women, which is associated with insulin resistance and type 2 diabetes mellitus (Haarbo et al., 1991). However, in another study the changes in body composition associated with menopause, with gain in fat mass and loss in lean mass, were not prevented by HRT combining estrogen, progesterone, and calcium (Aloi et al., 1995). Nevertheless, women in the postmenopausal state, undergoing HRT combining both estrogens and progesterone, have a similar prevalence of OSA when compared with premenopausal women, and a lower prevelance than in postmenopausal women without HRT (Shahar et al., 2003). Furthermore, it was shown that the exogenous application of progesterone in rats reduced the frequency of apneas recorded during sleep, an effect abolished by mifepristone, a progesterone and glucocorticoid receptor antagonist (Yamazaki et al., 2005). These effects agree with the lower levels of sex hormones—progesterone, estradiol, and 17-OH progesterone—in women with sleep apnea (Netzer et al., 2003) and with the effects of HRT in OSA in post-menopausal women and suggest that HRT might have a significant role in the prevention or treatment of OSA in women. Also, in men testosterone levels tend to significantly increase after weight loss, and this could play a role in favoring weight maintenance in men (Pasquali et al., 1988). In fact, testosterone supplementation in both hypogonadal and obese men has been found to selectively reduce visceral fat and improve lipid abnormalities and insulin sensitivity at the same time that it improves OSA-related dysfunctions (Gambineri and Pasquali, 2000).

Regarding insulin sensitivity impact, a lot of controversy is also present in the literature mainly due to the routes of administration of hormones since orally administered HRT is subject to first-pass hepatic metabolism, unlike the transdermal route of administration. Also, the different methods used to assess antidiabetic actions of HRT can justify the discrepancies in the results described for the use of this type of therapy. For example, oral estrogen replacement consisting of 2 mg of oral estradiol plus 1 mg/day of norethisterone decreased blood glucose, c-peptide, total cholesterol, and low-density lipoprotein showing a clear improvement in insulin action and carbohydrate homeostasis (Anderson et al., 1997). Yet, another study using transdermal estradiol treatment in comparison to oral conjugated estrogens showed that transdermal HRT did not alter insulin sensitivity or glucose tolerance but oral administration resulted in a deterioration of glucose metabolism (Godsland et al., 1993).

Some of the adverse effects of HRT on carbohydrate metabolism and insulin sensitivity have resulted from the use of higher doses of estrogens and the use of progestogens with higher androgenic effects. Progestogen-alone formulations containing levonorgestrel and medroxyprogesterone are associated with deterioration of glucose tolerance, whereas norethisterone has little effect. Also, the use of estradiol and dydrogesterone in oral formulations seems to avoid undesirable effects on the lipid metabolism (Foster and Balfour, 1997).

The mechanisms involved in the impact of HRT over metabolism in the context of OSA are not well understood and more research is needed. From the ones already detected we can find the impact of HRT in decreasing visceral fat deposition by increasing lipid oxidation and enhancing energy expenditure (Kim et al., 2014), in improving glucose homeostasis, and insulin sensitivity by the direct action of estrogens in estrogen receptors in the liver, skeletal muscle, adipose tissue or pancreatic cells (Tiano and Mauvais-Jarvis, 2012; Mauvais-Jarvis, 2016), or by preventing the increase in fasting glucose and insulin levels due to the impact on hepatic glucose production and hepatic insulin clearance (Kim et al., 2014).

Overall, since the beginning of HRT application in clinical practice, different and contrary opinions have been raised in the literature, but nowadays the general opinion of the scientific and medical communities is that HRT should be used with the smallest hormone dose possible and taking into account the comorbidities of each patient.

### Other Therapies With Impact Over OSA and Dysmetabolism

Besides CPAP and HRT, there are other therapies for OSA, comprising mandibular repositioning splints (MRS) and therapies with low application in clinical practice such as hypoglossal nerve stimulation and surgery. These therapies have been assessed in both women and men, although the literature lacks studies involving the female population (Garg et al., 2016; Wray and Thaler, 2016). However, two studies have suggested that MRS use is more efficient in women compared to men (Marklund et al., 2004; Vecchierini et al., 2019). In fact, obese men or men that gain weight during MRS treatment exhibit a reduced efficacy of their treatment with this device, and were often followed up with other OSA treatments like CPAP.

Nutritional and behavioral treatments to achieve weight loss have also been seen as beneficial in improving respiratory function (Pasquali et al., 1988) and metabolic abnormalities (Muscelli et al., 1997) with a higher extent for men than women (Newman et al., 2005).

## Conclusion

While there are clear sex differences in the pathological mechanisms, symptoms presentation, and disease manifestation in the link between OSA and dysmetabolism, more information needs to be unveiled to better understand sex-related phenotypes in OSA as well as to develop diagnostic tools for OSA in women.

## Data Availability Statement

The original contributions presented in the study are included in the article/supplementary material, further inquiries can be directed to the corresponding author/s.

## Author Contributions

FM and SC wrote or contributed to the writing of the manuscript. The authors have approved the final version of the manuscript, and all persons designated as authors qualify for authorship, and all those who qualify for authorship are listed.

## Funding

The author FM is funded by the Portuguese Foundation for Science and Technology with contract reference CEECIND/04266/2017.

## Conflict of Interest

The authors declare that the research was conducted in the absence of any commercial or financial relationships that could be construed as a potential conflict of interest.

## Publisher's Note

All claims expressed in this article are solely those of the authors and do not necessarily represent those of their affiliated organizations, or those of the publisher, the editors and the reviewers. Any product that may be evaluated in this article, or claim that may be made by its manufacturer, is not guaranteed or endorsed by the publisher.
